# Molecular dynamics simulation of nanoindentation on Cu/Ni nanotwinned multilayer films using a spherical indenter

**DOI:** 10.1038/srep35665

**Published:** 2016-10-21

**Authors:** Tao Fu, Xianghe Peng, Xiang Chen, Shayuan Weng, Ning Hu, Qibin Li, Zhongchang Wang

**Affiliations:** 1 College of Aerospace Engineering, Chongqing University, Chongqing 400044, China; 2 State Key Laboratory of Coal Mine Disaster Dynamics and Control, Chongqing University, Chongqing 400044, China; 3 Advanced Manufacturing Engineering, Chongqing University of Posts and Telecommunications, Chongqing 400065, China; 4 Advanced Institute for Materials Research, Tohoku University, 2-1-1 Katahira, Aoba-ku, Sendai 980-8577, Japan

## Abstract

We performed molecular dynamics simulation of nanoindentation on Cu/Ni nanotwinned multilayer films using a spherical indenter, aimed to investigate the effects of hetero-twin interface and twin thickness on hardness. We found that both twinning partial slip (TPS) and partial slip parallel with twin boundary (PSPTB) can reduce hardness and therefore should not be ignored when evaluating mechanical properties at nanoscale. There is a critical range of twin thickness *λ* (~25 Å < *λ* < ~31 Å), in which hardness of the multilayer films is maximized. At a smaller *λ*, TPSs appear due to the reaction between partial dislocations and twin boundary accounts for the softening-dominated mechanism. We also found that the combination of the lowered strengthening due to confined layer slips and the softening due to TPSs and PSPTBs results in lower hardness at a larger *λ*.

Metallic multilayered films and their mechanical properties have become a hot research topic over the past decades because of their promising technological applications and fundamental scientific importance^1^,^2^,^3^,^4^,^5^. To date, much effort has been devoted to unveil potential mechanisms related to the excellent mechanical performance of the multilayered films. Among these films, copper-nickel (CuNi) films have captured a great deal of attentions due to their small lattice mismatch and excellent properties^6^,^7^,^8^, and the embedded interfaces are thought to be one of the most important factors affecting the properties. It has been conjectured that incoherent interfaces may act as strong barriers to restrain dislocations from transmitting across the interfaces^9^ and also confine dislocations within each layer^10^,^11^,^12^. By using a high-resolution transmission electron microscopy, nanotwinned coherent interfaces have been observed in (111) textured Cu/Ni multilayer films^13^. Similar twin structures have also been detected in Cu/Ni multilayer films^2^. However, the strengthening and hardening mechanisms of such nano-multilayer films remain unclear, mainly because one of the key potential factors, the nanotwinned interface, is rarely known despite of its critical importance in affecting the mechanical properties of the films.

In recent years, much effort has been made to probe the effects of nanotwinned boundary on mechanical behaviors of nano-multilayer films. Li *et al*. performed a large-scale molecular dynamics (MD) simulation of uniaxial tension of nanotwinned polycrystalline Cu^14^, and suggested a dislocation-nucleation-controlled softening mechanism in nanotwinned metals, i.e. when the twin thickness is below a threshold value, the nucleation of dislocation may result in softening, governing the strength of a material. However, it is not clear if this kind of mechanism could be extended to Cu/Ni nanotwinned multilayer films. Zhu *et al*.^15^ simulated the uniaxial tension of a Cu/Ni nanotwinned multilayer film, and concluded that hairpin-like dislocation gliding dominates its inelastic deformation if the twin thickness is large, but multiple jogged necklace-like dislocations become dominant in the film with thin twin lamellae. Although the two kinds of views are different^14^,^15^, they both highlighted the importance of the reaction between partial dislocations and twin boundaries.

Shao *et al*. investigated the strengthening effects of semi-coherent interfaces^7^ and coherent interfaces^16^ in Cu/Ni multilayer films with MD simulations. Zhao *et al*.^17^ studied the effects of misfit dislocation network and modulation period on the hardness of Ag/Ni multilayer films. These studies help us to gain insights into the effects of interface on the strengthening of multilayer films; nevertheless, the softening mechanism and the effect of modulation period on hardness of Cu/Ni multilayer films can hardly be found in the literature. Recently, some simulations have been performed on the nanoindentation of Cu/Ni multilayer films with cylindrical indenters, revealing that the nanotwinned interface may improve the hardness of the multilayer films^18^, but the effect of twin thickness, especially the softening effect at small twin thickness requires an in-depth investigation. On the other hand, a cylindrical indenter was used to conduct nanoindentation with a two dimensional (2D) specimen in the previous work, in which the in-plane dislocation reaction may be ignored and the evolution of defects mat be constrained. Therefore, a realistic three dimensional indentation is needed. Nanoindentaion simulation with a sharp, conical or pyramidal indenter involves very complex problems. For example, stress–strain relationships may not be uniquely determined with the loading and unloading curves alone if a conical or pyramidal indenter is used, because of its geometric self-similarity^19^. Therefore, the simplest spherical indenter has widely been used to perform nanoindentation in MD simulations^20^,^21^.

In this article, we performed MD simulations of nanoindentation on Cu/Ni nanotwinned multilayer films with various twin thicknesses using a spherical indenter, aimed to investigate the reaction between the activated dislocations and nanotwinned interfaces under three-dimensional stress states. Moreover, the effects of twin thickness on the strengthening and hardening of the Cu/Ni multilayer films were also analyzed in detail.

## Methods

The embedded atom method (EAM) potential developed by Daw and Baskes^22^,^23^ was chosen to describe the force between the atoms in the multilayer films, involving Cu-Cu, Ni-Ni and Cu-Ni interatomic potentials. The parameters for the potentials were given by Zhou and Wadley^24^, which had been validated to be able to reproduce the physical properties of Cu-Ni system^25^. The Morse potential^26^, which had widely been used to solve surface contact problems^27^,^28^, was adopted to compute the interaction between the atoms in the indenter and multilayer film (C-Ni and C-Cu potentials), and the parameters involved were taken from the works by Chang *et al*.^29^ and Imran *et al*.^30^. Compared with the deformation in metallic multilayer films, the deformation of the indenter could be ignored, i.e. the indenter was assumed rigid^31^. The motion of all the atoms in the indenter was neglected to reduce computation time^32^.

**Figure 1:**
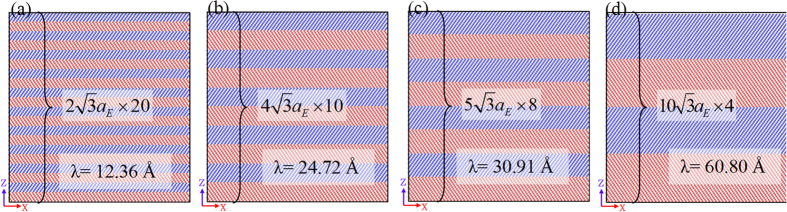
Initial configurations of the samples viewed on the X-Z plane with different twin thichness: (**a**) 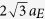
(12.36 Å), (**b**) 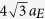
(24.72 Å), (**c**) 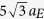
(30.91 Å) and (**d**) 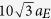
(61.80 Å). Red and blue balls represent Cu and Ni atoms, respectively.

Since the FCC metallic multilayer films are frequently found to grow mainly along <111> orientations^13^,^33^, we took the {111} plane as the indentation surface. We introduced nanotwinned interfaces into the multilayer films, and let the *X, Y* and *Z* axes correspond to lattice orientations of [112], 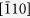 and 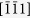 for Cu layers, and of 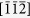, 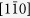 and 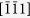
for Ni layers. Figure 1 showed an initial configuration of the Cu/Ni multilayer films on the *X-Z* plane of different twin thickness. The lattice constant of single Cu (*a*_Cu_) at 0 K was 3.615 Å and that of Ni (*a*_Ni_) was 3.520 Å, hence, the lattice misfit between Ni and Cu was ~2.7%. To build the samples with coherent twin interface, an average lattice constant, *a*_*E*_ = (*a*_Cu_ + *a*_Ni_)/2, was set as the lattice constant of Cu and Ni to arrange atoms. Hence, ε_*xx*_ = ε_*yy*_ = (*a*_*E*_—*a*_Cu_)/*a*_Cu_ = −0.0131(ε) were imposed upon the Cu, layers, and ε_*xx*_ = ε_*yy*_ = (*a*_E_—*a*_Ni_)/*a*_Ni_ = 0.0134 were imposed upon the Ni layers to accommodate the misfit lattice. The *X* and *Y* lengths of the samples were 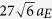
(27 <112> *a*_*E*_ or 235.94 Å) and 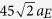
(45 <110> *a*_*E*_ or 227.03 Å), respectively. For the initial configuration, the thickness of Cu and Ni layers was the same, i.e. the modulation ratio was 1. Since the twin thickness (*λ*) was defined as the spacing between two adjacent twin boundaries, the thickness of a single Cu (or Ni) layer was also twin thickness. The height of the samples (Z direction) was 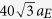
(40 <111> *a*_*E*_ or 247.16 Å). To make sure that the samples possess the same atoms of Cu and Ni, the equation, (*λ*_Cu_ + *λ*_Ni_) × *n* = l_z_ = 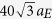, should be satisfied. Because *λ*_Cu_ = *λ*_Ni_ and *n* is an integer, the twin thickness (*λ*_Cu_ = *λ*_Ni_) can be set as 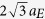
(12.36 Å), 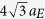
(24.72 Å), 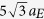
(30.91 Å) and 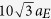
(61.80 Å), corresponding to *n* = 10, 5, 4, 2, respectively. Figure 1 shows the four samples labeled with A, B, C and D, corresponding to different thicknesses of 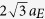,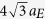, 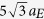 and 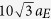, respectively. Each sample contains 1,166,400 atoms.

**Figure 2:**
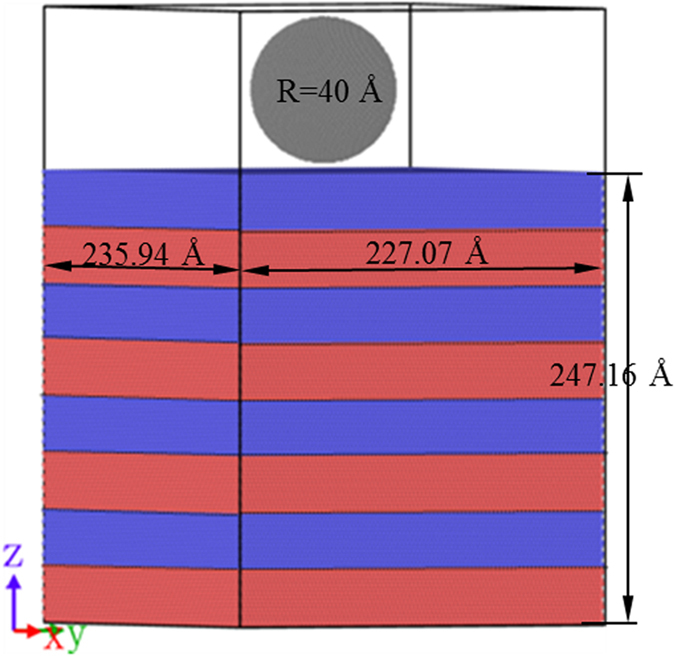
Schematics of the indentation model. Red, blue and grey balls represent Cu, Ni and C atoms, respectively.

Figure 2 showed an initial configuration of Sample C for the nanoindentation. During the indentation, the atoms in the three layers at the bottom were fixed preventing substrate from shifting. These atoms served as the boundary atoms. The rest atoms were fixed at a constant temperature by the Langevin thermostat^34^ as thermostat atoms. The motion of the thermostat atoms followed the classical Newton’s second law, and hence these atoms were called Newtonian atoms. The conjugate gradient (CG) algorithm was used for energy minimization. To better understand the reaction between interface and deformation induced defect, in the case of low temperature at which the effect of the random vibration of atoms can be ignored, simulations of indentation were performed at 10 K. Periodic boundary condition was imposed in both X and Y directions. Before indentation, each sample was relaxed at 10 K for 20 ps to reach a thermal equilibrium state. Based on the explorations about indenter size^35^,^36^,^37^ and indentation speed^35^,^36^,^38^,^39^, a spherical diamond indenter with a diameter of 80 Å was used and the indentation speed was 50 m/s. The maximum indentation depth was 32 Å, smaller than the radius of the indenter and close to the twin thickness of Sample C.

The dislocation extraction algorithm (DXA) was used to analyze local disorder. It could not only divide the atoms into different types of local structures (FCC, BCC, HCP, etc.), but also identify all dislocations in FCC crystal, determine their Burgers vectors, and output lines that represent dislocations^40^. The colors of an atom represented the local lattice structure of the atom and were assigned as: green for FCC, red for HCP, and white for “other” local crystal structures. A single red layer of atoms indicated a twin boundary (TB), while two adjacent red layer atoms represented a stacking fault (SF). The open software OVITO^41^ was used to color the atoms with different local lattice structures.

## Results and Discussion

### Deformation mechanism of the Cu/Ni multilayer films

Before performing the nanoindentation on a Cu/Ni multilayer film, we demonstrated the validity of the potential and the nanoindentation model by comparing the fundamental physical properties predicted with MD simulation with that obtained with first principles calculation/experiment (Tables S1 and S2 in Supplementary Materials), and comparing the simulation result of the indentations on pure Cu and Ni films with the result by Hertz solution (Figure S1 in Supplementary Materials). From Tables S1 and S2 in Supplementary Materials, we can see that these potentials can be used in the simulations for Cu-Ni systems. By comparing the indentation force-depth (*P*-*h*) curve obtained with MD simulation with that with Hertz theory, we can find that they matches each other with an acceptable error (Figure S3 in Supplementary Materials), especially, the *P*-*h* curves obtained using an perfectly smooth sphere realized by a repulsive potential well matches the Hertz theory (Figure S3 in Supplementary Materials). We also performed the nanoindentation simulations on Cu and Ni films with different indentation speeds, and found that the fitted “reduced modulus”, *E*, of the Hertz theory decreases with the decrease of indentation speed, and the *E* obtained at indentation speed of 1 m/s matched the theoretical solution better than that at larger indentation speeds^20^ (Figure S3 in Supplementary Materials). The depths at the first peaks of the *P-h* curves corresponding to different indentation speeds for Cu or Ni were very close to each other, indicating that that the effects of the speed range (*v* = 1 m/s to *v* = 50 m/s) are insufficient for the study of the deformation mechanisms of the films under nanoindentation. However, lower indentation velocity will spend much more computational time than higher indentation velocity. For example, it would spend fifty times computational time with 1 m/s than that with 50 m/s. Therefore, considering the computational efficiency and the limitation of computational capability, the indentation velocities range from 10 m/s to 100 m/s were commonly chosen to perform MD simulations^35^,^36^,^38^,^39^. Therefore, the indentation velocity of 50 m/s was selected to carry out MD simulations in this work.

**Figure 3:**
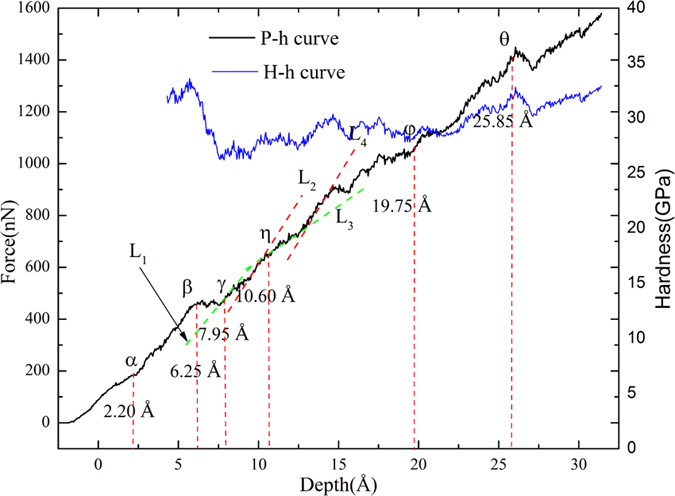
*P-h* and *H-h* curves for the Sample C with indenter size of 40 Å in radius.

**Figure 4:**
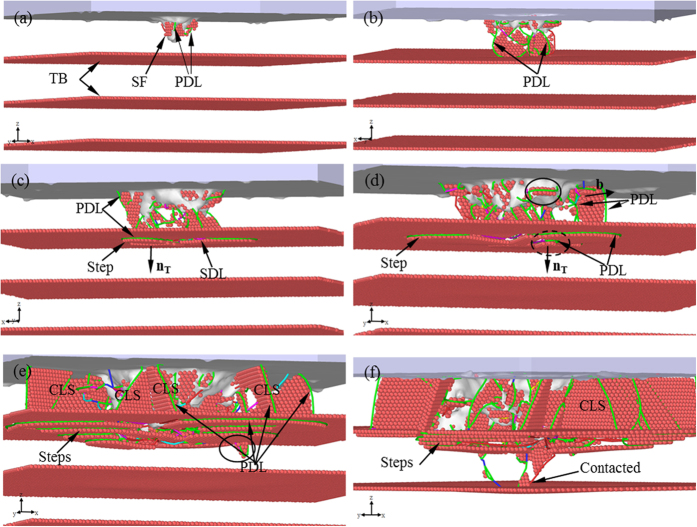
Microstructural evolution of the Sample C as a function of *h*. (**a**) *h* = 2.20 Å, (**b**) *h* = 6.25 Å, (**c**) *h* = 7.95 Å, (**d**) *h* = 10.60 Å, (**e**) *h* = 19.75 Å, and (**f**) *h* = 25.85 Å.

**Figure 5:**
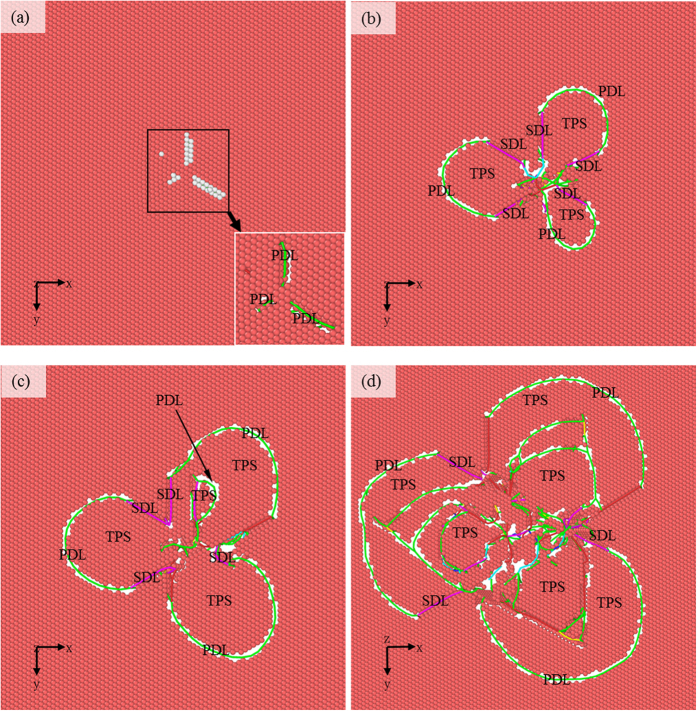
Bottom view of the first TB. The atoms in local HCP lattice structure are colored. Green and pink lines represent the 1/6 <112> Shockley partial dislocation lines (SPDLs) and 1/6 <110> stair-rod dislocation lines (SDLs), respectively. The twinning partial slips (TPSs) are surrounded by dislocation lines.

The sample C is chosen to investigate the reaction between dislocations and nanotwinned interface, in which the twin thickness is close to the maximum indentation depth. Figure 3 shows the *P-h* curve, where there are many drops or fluctuations, corresponding to different plastic deformation mechanisms. To clarify the deformation mechanisms, we present in Fig. 4(a–f) the microstructures at characteristic points α, β, γ, η, φ and θ in the *P*-*h* curve. The atoms in the figure identified as FCC and “other” lattice structure as well as the indenter atoms have been removed for clarity. At Point α (Fig. 3), some slight fluctuations can be observed. These fluctuations are not apparent compared with that caused by the reaction between dislocation and twin interface. To observe these fluctuations more clearly, the local *P-h* curve at Point α is amplified and shown in Figure S4(a) in Supplementary Materials. Accordingly, it can be seen in Fig. 4(a) that some Shockley partial dislocations (SPDs) nucleate (the green lines represent Shockley partial dislocation lines (SPDLs)) and extend, forming inclined stacking faults (SFs). Therefore, Point α can be regarded as the transition point from elastic to plastic deformation, which coincides with the simulation results by Lilleodden, *et al*.^42^ and Lee, *et al*.^43^. At Point β when *h* = 6.25 Å, one can still note some slight fluctuations in the *P-h* curve, indicating nucleation of new SPDs, and the amplified local *P*-*h* curve near Point β is shown in Figure S4(b). Li *et al*. proposed that dislocation nucleation results in softening of polycrystalline materials^14^, which has also been found in Cu/Ni nanotwinned multilayer films under indentation with a cylindrical indenter^18^. In Fig. 3, the *P-h* curve exhibits a persistent ascending tendency with no distinct drop between Points α and β, as was also seen in the indentation of Ag/Ni multilayer films with a spherical indenter^17^, indicating that the softening induced by partial dislocation nucleation is insignificant. Figure 4(b) shows the microstructure of the sample at Point β, where more SPDs nucleate, some of which even propagate to the first interface (or the first twin boundary (TB)). Figure 5(a) shows the bottom view of the first TB, where one can see that, (1) the surface keeps flat without distinct convexities, and (2) some atoms are indentified as “other” structure (Fig. 5(a)). After removing the atoms in “other” structure, three segments of SPDLs appear, implying no reaction between the SPDs and TB. These atoms can be ascribed to the change of the surrounding environment of TB atoms with SPDs adjoining to the TB. It should be noted that all the SPDLs here are on the inclined slip plane rather than on the plane parallel with the TB (Fig. 4(b)).

By comparing the microstructure at Point γ (Fig. 4(c)) with Point β (Fig. 4(b)), one can find that: (1) some new SPDs nucleate forming inclined slips; (2) some TB atoms surrounded by three semicircular dislocation lines (DLs) move downwards, forming twinning partial slips. Figure 5(b) shows the bottom view of the TB in Fig. 4(c), where it can be seen that the three semicircular dislocation lines consist of several segments of 1/6 <112> SPDs and 1/6 <110> stair-rod dislocation lines (SDLs). The presence of SDLs indicates either nucleation of new dislocations from TB or reaction between dislocations and TB. The detailed discussion on the reaction process to form step or stair-rod dislocations can be found in our previous work^18^ or the theoretical derivation by Zhu, *et al*.^44^. The segment between Points β and γ is almost horizontal (Fig. 3), implying the softening of the material. This distinct softening should mainly be ascribed to the formation of twinning partial slips^45^.

The indentation force *P* ascends with the increase of *h* due to the increase of the contact area even if no softening or strengthening is considered. To better analyze these effects, the hardness *H*, which is defined as the ratio of *P* to the projected contact area *S*^46^, i.e. *H* = *P*/*S*, can eliminate the effect due to the increase of area, where *S = π*(2*R*-*h*_*c*_)*h*_*c*_^47^, with *R* and *h*_*c*_ the indenter radius and contact depth, respectively. Since the indenter surface is unsmooth and extraction of contact depth is difficult, the projected contact area is usually calculated with *S = π*(2*R*-*h*)*h*^47^,^48^ in plastic deformation region, as was mentioned by Li *et al*., that “small differences in the resulting hardness is expected but it would not be large enough to change the general picture and conclusion of this work”^48^. The *H-h* curve after elastic deformation is also shown in Fig. 3, where softening can be further confirmed in the part between Points β and γ.

At the stage between Points γ and η, both *P-h* and *H-h* curves ascend with the increase of *h*, implying that some other mechanisms may play a role apart from the softening effect. The main microstructural changes in this stage are that, (1) horizontal TPSs extend, indicating the existence of softening; and (2) some slip planes inclined to TB and the Burgers vector **b** parallel with TB form, resulting in the slips of dislocations confined within a single layer (we call it confined-layer slip, CLS)^45^. It can be seen in Fig. 3 that the slope of L_2_ is larger than that of L_1_, which is attributed to the CLS induced strengthening effect. A new reaction between dislocation and TB occurs (see the dashed-line ellipse in Fig. 4(d)) and the TPSs extend, imposing subsequent softening and thus resulting in a low slope of L_3_ and hardness reduction. With the further increase of *h*, partial dislocations nucleate and then CLSs develop, enhancing the CLS strengthening, which account for the larger slope of L_4_ compared with that of L_3_ and the increase of hardness. Figure 4(e) shows the microstructure at Point φ (Fig. 3), where many partial dislocations (PDs) nucleate and move, forming inclined slips. Most of them have the Burgers vector **b** that is paralleled with TB. The corresponding bottom view of the TB at Point φ is shown in Fig. 5(d), where the three semicircular DLs further expand and some new DLs appear, demonstrating the extension of the TPSs resulting from the reaction between PDs and TB, as was also confirmed by the terrace shown in Fig. 4(e). In the range of 15 Å < *h* < 19.75 Å, many fluctuations can be observed in the *P-h* curve due to the competition between softening and CLS strengthening. It can be seen in the ellipse in Fig. 4(e) that a PD crosses the TB. Before *h* = 25.85 Å (Point θ), the number of inclined slips that cross the TB increases slowly and only few ones contact the next TB (Fig. 4(f)), indicating that the CLS strengthening prevails when competed with softening. A drop appears after Point θ, which is attributed to the reaction between PD and TB and the formation of TPSs, which contributes to softening.

### Effects of twin thickness

**Figure 6:**
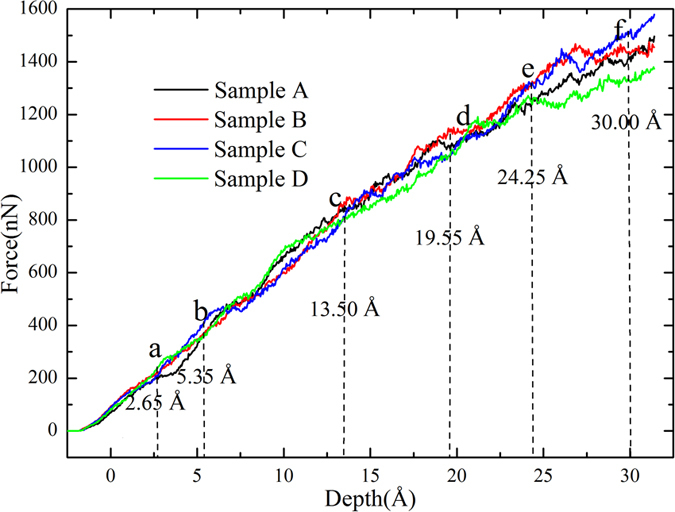
*P-h* curves of the samples with various twin thicknesses with indenter size of 40 Å in radius.

**Figure 7:**
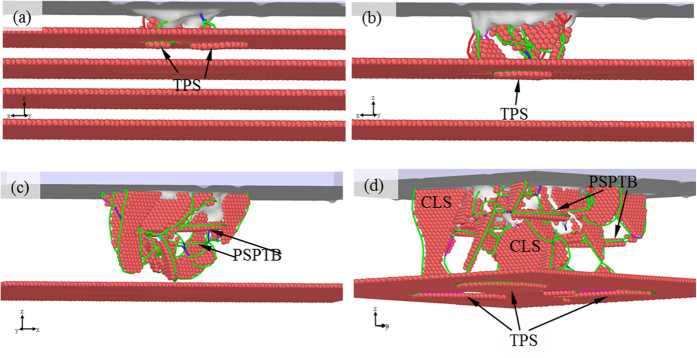
Microstructures of the samples at different *h*. (**a**) Sample A at *h* = 2.65 Å; (**b**) Sample B at *h* = 5.35 Å; (**c**) Sample D at *h* = 13.50 Å; (**d**) Sample D at *h* = 19.55 Å.

The TPS softening mechanism and the confined layer slip strengthening mechanism have been preliminarily discussed in the previous section by analyzing the simulation result for Sample C. To further explore the effect of twin thickness on hardness, we also conducted MD simulations of indentation on Samples A, B and D. Figure 6 shows the *P-h* curves, which can be divided in general into Parts I, II, III, IV, V and VI. Before *h* = 2.45 Å, the four curves well match each other because of the insignificant effects of twin boundary on elastic response. With the increase of *h*, a slight plateau first appears in the *P-h* curve of Sample A, implying softening. Figure 7(a) shows the microstructure of sample A at Point a (*h* = 2.65 Å), in which it can be seen that the reaction between dislocations and TB occurs and TPSs form. In Part III, the softening due to TPSs dominates the deformation of Samples B and C, resulting in a low part in the *P-h* curves of the two samples. However, the difference in these two cases is not obvious since the twin thickness of Sample B is close to that of Sample C.

**Figure 8:**
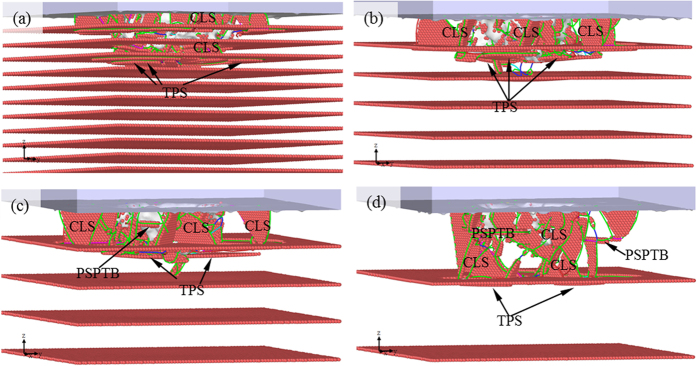
Microstructures of different samples at *h* = 24.25 Å. (**a**) Sample A, (**b**) Sample B, (**c**) Sample C, and (**d**) Sample D.

Figure 7(b) shows the microstructure of Sample B at *h* = 5.35 Å (Point b in Fig. 6), where one can note the formation of TPSs, accounting for softening. In Part IV, the *P-h* curve of Sample D becomes the lowest among the four. The microstructure for Sample D at *h* = 13.50 Å (Point c in Fig. 6) is shown in Fig. 7(c), where it can be seen that the partial dislocations do not contact TB, and the TPSs are not found, implying that in this case TPSs are not the main cause of softening. However, some horizontal partial slips can be found in Fig. 7(c), which are characterized as TPSs, of which both the slip plane and Burgers vector **b** are parallel with TB. The softening can thus be ascribed to these partial slips that are parallel with twin boundary (PSPTB). With the increase of *h*, the softening continues due to the movement of the PSPTBs and TPSs until Point d is reached. The confined layer slips are also observed (Fig. 7(d)) and the competition between softening and strengthening should be taken into account. In Part V, it becomes considerable, resulting in slight difference between the four curves. On the other hand, the role of new nucleation of PDs and the cross reaction between PDs in the layer should be insignificant. Figure 8 shows microstructures of different samples at *h* = 24.25 Å (Point e in Fig. 6), in which all the main kinds of slips can be found. After Point e, the difference between the four curves becomes obvious, implying different deformation mechanisms related to different twin thicknesses.

**Figure 9:**
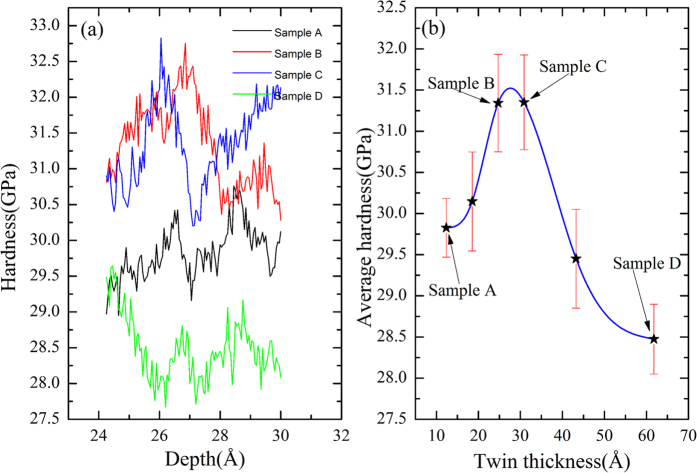
Variation of harness against *h* and *λ* for the samples with indenter size of 40 Å in radius: (**a**) *H-h* and (**b**) *H-λ* curve.

To further explore the effects of twin thickness, we calculated the hardness *H*. It can be seen that after Point e, all the main kinds of slips emerge in each sample (Fig. 8) and both strengthening and softening contribute to the property of each sample. The *H-h* curves for the four samples in Part VI are shown in Fig. 9(a). It should be noted that the simulated hardness is larger than the experimental one^2^, because (1) the time scale used in simulation differs from that used in experiment, resulting in a much larger indentation speed than that in experiment, (2) in-plane polycrystalline configuration is not considered in simulation, and (3) the temperature in simulation (10 K) is much lower than that in experiment. Despite of these differences, our simulated hardness is close to the simulated results by others^49^. One can find that Samples B and C have larger hardness than Samples A and D. For detailed analysis, the mean of hardness of the four samples with error bars are shown in Fig. 9(b), where it can be seen that the hardness of Samples B (*λ* = 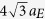) and C (*λ* = 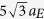) is much larger, while it decreases if either *λ* < 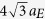 or *λ* > 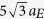. The twin thickness corresponding to maximum hardness should be 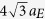 < *λ* < 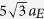, which agrees well with the experimental results^13^,^50^. For the validity of the results, two additional samples are prepared for the nanoindentation simulations. However, it is difficult to build two more different samples with identical overall size but the layer thickness is different from the ones that have been used. Hence, the thicknesses of the one or two bottom layers that are far away from the loaded layer are different from that of the upper layers, and the initial configurations of the two additional samples viewed on the X-Z plane are presented in Figure S5 in Supplementary Materials. The mean hardness at the depth range from 24.25 Å to 31 Å of these two additional samples with error bars are also calculated and shown in Fig. 9(b), where the six points are fitted with a spline. It can be seen that the maximum hardness appears in the range of twin thickness, *λ* (~25 Å < *λ* < ~31 Å). The hardness decreases with both the increase and decrease of *λ*, which is similar to the Hall-Petch and inverse Hall-Petch relation. Conventional Hall-Petch^51^ and inverse Hall-Petch relations^52^ describe the relation between the strength of a material and its grain size, but in this work, we used the Hall-Petch and inverse Hall-Petch relations for the relationship between the strength of a multilayer and its twin thickness. Therefore, some certain relationship may exist between the Hall-Petch-like/inverse Hall-Petch-like effect and the twin layer/thickness in multilayer films.

Making use of the result given in Fig. 9(b), we can simply evaluate the hardness of the multilayer films with


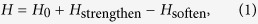


in which *H*_strenghten_ and *H*_soften_ correspond to the contributions from strengthening and softening effects, respectively. *H*_0_ represents the hardness without taking into account the strengthening and softening effects. In this work, strengthening is mainly related to confined layer slip effect (or dislocation blocking at interface) *H*_CLS_, while softening effect is mainly related to twinning partial slip *H*_TPS_, partial slip parallel with twin boundary *H*_PSPTB_, and nucleation of dislocation softening effect *H*_NDSF_. Thus, one can obtain


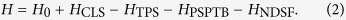


As was discussed in Subsection 3.1, *H*_NDSF_ can be ignored and *H*_CLS_ decreases with the increase of twin thickness^53^, i.e. *H*_CLS_ (Sample A) > *H*_CLS_ (Sample B) > *H*_CLS_ (Sample C) > *H*_CLS_ (Sample D). For Sample A, PSPTBs cannot form due to small twin thickness, i.e. *H*_PSPTB_ = 0. However, the TBs participating in the reaction are the primary ones with the same depths. It is known that more TBs may give rise to the more reaction between dislocation and TBs, resulting in the increase of TPSs. The softening induced by the TPSs plays a dominant role in the hardness of sample A.

The twin thickness of Sample B is small and PSPTB can hardly form, i.e. *H*_PSPTB_ = 0. Meanwhile, the number of TBs in Sample B is half of that in Sample A, so that softening is reduced obviously. Incorporating *H*_CLS_, *H*_CLS_-*H*_TPS_ may reach the largest. It can be seen in Fig. 8(b,c) that almost all the slips are confined within the top layer of Samples B and C. *H*_CLS_ of Sample B is comparable with that of Sample C. It can be seen in the solid-line ellipse in Fig. 4(d) that few PSPTBs appear and therefore *H*_PSPTB_ is nonzero. However, the effect of *H*_TPS_ decreases and the two effects counteract. The hardness of Sample C is thus comparable to that of Sample B. The twin thickness of Sample D is large and therefore *H*_CLS_ should be the smallest. Meanwhile, although *H*_TPS_ decreases with the increase of twin thickness, there are PSPTBs (Fig. 8(d)) and the combination of the two factors accounts for the lowest hardness of Sample D.

To guarantee the validity of the results, additional nanoindentation simulations are performed on the four samples (A, B, C and D) with a larger indenter of 50 Å in radius and a lower indentation speed of 10 m/s, and the similar result have been obtained. Related simulation results are shown in Figures S6–S9 of Supplementary Materials.

## Conclusions

We have performed MD simulations of indentation on Cu/Ni nanotwinned multilayer films with a spherical indenter, aimed to investigate the effects of the nanotwinned interface and the twin thickness on hardness. We find that the TPS and PSPTB can lower the hardness, which shall not be ignored in evaluating mechanical property on nanoscale. It is also found that there is a critical range for twin thickness, within which hardness reaches maximum or otherwise it is reduced. For small twin thickness, the TPS forms due to the reaction between the partial dislocations and twin boundary, which corresponds to softening mechanism. The combination of limited strengthening due to confined layer slips and enhanced softening due to the TPS and PSPTB can result in a smaller hardness at a larger twin thickness. These findings help to understand the strengthening and softening effects of nanotwinned multilayer films, which may have implications for some important phenomena, such as Hall-Petch effect and inverse Hall-Petch effect, despite of the strong dislocation blocking strengthening.

## Additional Information

**How to cite this article**: Fu, T. *et al*. Molecular dynamics simulation of nanoindentation on Cu/Ni nanotwinned multilayer films using a spherical indenter. *Sci. Rep.* **6**, 35665; doi: 10.1038/srep35665 (2016).

## Supplementary Material

Supplementary Information

## References

[b1] Zhang B., Kou Y., Xia Y. Y. & Zhang X. Modulation of strength and plasticity of multiscale Ni/Cu laminated composites. Mat Sci Eng a-Struct 636, 216–220 (2015).

[b2] Zhu X. Y., Liu X. J., Zong R. L., Zeng F. & Pan F. Microstructure and mechanical properties of nanoscale Cu/Ni multilayers. Materials Science and Engineering: A 527, 1243–1248 (2010).

[b3] Han W. Z. . Deformation and failure of shocked bulk Cu-Nb nanolaminates. Acta Materialia 63, 150–161 (2014).

[b4] Zheng S. . High-strength and thermally stable bulk nanolayered composites due to twin-induced interfaces. Nature communications 4, 1696 (2013). 10.1038/ncomms2651 23591863

[b5] Han W. Z., Carpenter J. S., Wang J., Beyerlein I. J. & Mara N. A. Atomic-level study of twin nucleation from face-centered-cubic/body- centered-cubic interfaces in nanolamellar composites. Applied Physics Letters 100, 011911 (2012).

[b6] Rao S. I. & Hazzledine P. M. Atomistic simulations of dislocation–interface interactions in the Cu-Ni multilayer system. Philosophical Magazine A 80, 2011–2040 (2000).

[b7] Shao S., Wang J., Beyerlein I. J. & Misra A. Glide dislocation nucleation from dislocation nodes at semi-coherent {1 1 1} Cu-Ni interfaces. Acta Materialia 98, 206–220 (2015).

[b8] Cammarata R. C., Schlesinger T. E., Kim C., Qadri S. B. & Edelstein A. S. Nanoindentation study of the mechanical properties of copper-nickel multilayered thin films. Applied Physics Letters 56, 1862 (1990).

[b9] Wang J., Hoagland R. G., Liu X. Y. & Misra A. The influence of interface shear strength on the glide dislocation- interface interactions. Acta Materialia 59, 3164–3173 (2011).

[b10] Misra A., Hirth J. P. & Kung H. Single-dislocation-based strengthening mechanisms in nanoscale metallic multilayers. Philosophical Magazine A: Physics of Condensed Matter, Structure, Defects and Mechanical Properties 82, 2935–2951 (2002).

[b11] Misra A., Hirth J. P. & Hoagland R. G. Length-scale-dependent deformation mechanisms in incoherent metallic multilayered composites. Acta Materialia 53, 4817–4824 (2005).

[b12] Wang J. & Misra A. An overview of interface-dominated deformation mechanisms in metallic multilayers. Current Opinion in Solid State and Materials Science 15, 20–28 (2011).

[b13] Liu Y., Bufford D., Wang H., Sun C. & Zhang X. Mechanical properties of highly textured Cu/Ni multilayers. Acta Materialia 59, 1924–1933 (2011).

[b14] Li X., Wei Y., Lu L., Lu K. & Gao H. Dislocation nucleation governed softening and maximum strength in nano-twinned metals. Nature 464, 877–880 (2010). 20376146 10.1038/nature08929

[b15] Zhu Y. X., Li Z. H., Huang M. S. & Liu Y. Strengthening mechanisms of the nanolayered polycrystalline metallic multilayers assisted by twins. International Journal of Plasticity 72, 168–184 (2015).

[b16] Shao S. & Medyanik S. N. Dislocation–interface interaction in nanoscale fcc metallic bilayers. Mechanics Research Communications 37, 315–319 (2010).

[b17] Zhao Y. B. . MD simulation of nanoindentation on (001) and (111) surfaces of Ag-Ni multilayers. Physica E-Low-Dimensional Systems & Nanostructures 74, 481–488 (2015).

[b18] Fu T. . Molecular dynamics simulation of effects of twin interfaces on Cu/Ni multilayers. Materials Science and Engineering: A 658, 1–7 (2016).

[b19] Bucaille J. L., Stauss S., Felder E. & Michler J. Determination of plastic properties of metals by instrumented indentation using different sharp indenters. Acta Materialia 51, 1663–1678 (2003).

[b20] Zhu T. Predictive modeling of nanoindentation-induced homogeneous dislocation nucleation in copper. Journal of the Mechanics and Physics of Solids 52, 691–724 (2004).

[b21] Begau C., Hartmaier A., George E. P. & Pharr G. M. Atomistic processes of dislocation generation and plastic deformation during nanoindentation. Acta Materialia 59, 934–942 (2011).

[b22] Foiles S. M., Baskes M. I. & Daw M. S. Embedded-atom-method functions for the fcc metals Cu, Ag, Au, Ni, Pd, Pt, and their alloys. Physical Review B 33, 7983–7991 (1986). 10.1103/physrevb.33.7983 9938188

[b23] Johnson R. A. Alloy models with the embedded-atom method. Physical review. B, Condensed matter 39, 12554–12559 (1989). 10.1103/physrevb.39.12554 9948120

[b24] Zhou X. W. & Wadley H. N. G. Atomistic simulations of the vapor deposition of Ni/Cu/Ni multilayers: The effects of adatom incident energy. Journal of Applied Physics 84, 2301–2315 (1998).

[b25] Fu T. . MD simulation of effect of crystal orientations and substrate temperature on growth of Cu/Ni bilayer films. Applied Physics a-Materials Science & Processing 122, 1–9 (2016).

[b26] Morse P. M. Diatomic Molecules According to the Wave Mechanics. II. Vibrational Levels. Physical Review 34, 57–64 (1929).

[b27] Li J., Fang Q., Liu Y. & Zhang L. A molecular dynamics investigation into the mechanisms of subsurface damage and material removal of monocrystalline copper subjected to nanoscale high speed grinding. Applied Surface Science 303, 331–343 (2014).

[b28] Li J., Fang Q., Zhang L. & Liu Y. Subsurface damage mechanism of high speed grinding process in single crystal silicon revealed by atomistic simulations. Applied Surface Science 324, 464–474 (2015).

[b29] Chang W.-Y., Fang T.-H., Lin S.-J. & Huang J.-J. Nanoindentation response of nickel surface using molecular dynamics simulation. Molecular Simulation 36, 815–822 (2010).

[b30] Imran M., Hussain F., Rashid M. & Ahmad S. A. Molecular dynamics study of the mechanical characteristics of Ni/Cu bilayer using nanoindentation. Chinese Physics B 21, 126802 (2012).

[b31] Komanduri R., Ch, rasekaran N. & Raff L. M. Molecular dynamics simulation of the nanometric cutting of silicon. Philosophical Magazine Part B 81, 1989–2019 (2001).

[b32] Fu T. . Molecular dynamics simulation of deformation twin in rocksalt vanadium nitride. Journal of Alloys and Compounds 675, 128–133 (2016).

[b33] Liu Y. . A formation mechanism for ultra-thin nanotwins in highly textured Cu/Ni multilayers. Journal of Applied Physics 111, 073526 (2012).

[b34] Schneider T. & Stoll E. Molecular-dynamics study of a three-dimensional one-component model for distortive phase transitions. Physical Review B 17, 1302–1322 (1978).

[b35] Yang B. . Atomistic simulation of nanoindentation on incipient plasticity and dislocation evolution in γ/γ′ phase with interface and void. Computational Materials Science 114, 172–177 (2016).

[b36] Alhafez I. A., Ruestes C. J., Gao Y. & Urbassek H. M. Nanoindentation of hcp metals: a comparative simulation study of the evolution of dislocation networks. Nanotechnology 27, 045706 (2016). 26655887 10.1088/0957-4484/27/4/045706

[b37] Lu Z., Chernatynskiy A., Noordhoek M. J., Sinnott S. B. & Phillpot S. R. Nanoindentation of Zr by molecular dynamics simulation. Journal of Nuclear Materials 467, 742–757 (2015).

[b38] Fang T.-H., Chang W.-Y. & Huang J.-J. Dynamic characteristics of nanoindentation using atomistic simulation. Acta Materialia 57, 3341–3348 (2009).

[b39] Hasnaoui A., Derlet P. M. & Van Swygenhoven H. Interaction between dislocations and grain boundaries under an indenter – a molecular dynamics simulation. Acta Materialia 52, 2251–2258 (2004).

[b40] Stukowski A., Bulatov V. V. & Arsenlis A. Automated identification and indexing of dislocations in crystal interfaces. Modelling and Simulation in Materials Science and Engineering 20, 085007 (2012).

[b41] Stukowski A. Structure identification methods for atomistic simulations of crystalline materials. Modelling and Simulation in Materials Science and Engineering 20, 045021 (2012).

[b42] Lilleodden E. T., Zimmerman J. A., Foiles S. M. & Nix W. D. Atomistic simulations of elastic deformation and dislocation nucleation during nanoindentation. Journal of the Mechanics and Physics of Solids 51, 901–920 (2003).

[b43] Lee Y., Park J. Y., Kim S. Y., Jun S. & Im S. Atomistic simulations of incipient plasticity under Al(111) nanoindentation. Mechanics of Materials 37, 1035–1048 (2005).

[b44] Zhu Y. T. . Dislocation-twin interactions in nanocrystalline fcc metals. Acta Materialia 59, 812–821 (2011).

[b45] You Z. . Plastic anisotropy and associated deformation mechanisms in nanotwinned metals. Acta Materialia 61, 217–227 (2013).

[b46] Liu C. L., Fang T. H. & Lin J. F. Atomistic simulations of hard and soft films under nanoindentation. Mat Sci Eng a-Struct 452, 135–141 (2007).

[b47] Zhu P. Z. & Fang F. Z. Molecular dynamics simulations of nanoindentation of monocrystalline germanium. Applied Physics A 108, 415–421 (2012).

[b48] Li Y. . Nanoindentation of gold and gold alloys by molecular dynamics simulation. Materials Science and Engineering: A 651, 346–357 (2016).

[b49] Saraev D. & Miller R. E. Atomic-scale simulations of nanoindentation-induced plasticity in copper crystals with nanometer-sized nickel coatings. Acta Materialia 54, 33–45 (2006).

[b50] Schweitz K. O., Chevallier J., B⊘ttiger J., Matz W. & Schell N. Hardness in Ag/Ni, Au/Ni and Cu/Ni multilayers. Philosophical Magazine A 81, 2021–2032 (2001).

[b51] Pande C. S. & Cooper K. P. Nanomechanics of Hall-Petch relationship in nanocrystalline materials. Progress in Materials Science 54, 689–706 (2009).

[b52] Hahn E. N. & Meyers M. A. Grain-size dependent mechanical behavior of nanocrystalline metals. Materials Science and Engineering: A 646, 101–134 (2015).

[b53] Zhu T. & Gao H. J. Plastic deformation mechanism in nanotwinned metals: An insight from molecular dynamics and mechanistic modeling. Scripta Mater. 66, 843–848 (2012).

