# Supplementary material for: Molecular dynamics simulation of nanoindentation on Cu/Ni nanotwinned multilayer films using a spherical indenter
Source: Sci Rep. 2016 Oct 21;6:35665. doi: 10.1038/srep35665 (PMC5073369; doi:10.1038/srep35665)
Supplement: Supplementary Information [file srep35665-s1.pdf]

Supplementary materials

Molecular dynamics simulation of nanoindentation on Cu/Ni nanotwinned multilayer films using a spherical indenter

Tao Fu[1], Xianghe Peng[1, 2,*], Xiang Chen[3], Shayuan Weng[1], Ning Hu[1], Qibin Li[1], Zhongchang Wang[1, 4,*]

[1]*College of Aerospace Engineering, Chongqing University, Chongqing 400044, China*
[2]*State Key Laboratory of Coal Mine Disaster Dynamics and Control, Chongqing University, Chongqing 400044, China*
[3]*Advanced Manufacturing Engineering, Chongqing University of Posts and Telecommunications, Chongqing 400065, China*
[4]*Advanced Institute for Materials Research, Tohoku University, 2-1-1 Katahira, Aoba-ku, Sendai 980-8577, Japan*

***Corresponding authors.**

Address: College of Aerospace Engineering, Chongqing University, Chongqing 400044, China

TEL: +86–23–65103755; FAX: +86–23–65102521

E-mails: xhpeng@cqu.edu.cn (X.P.); zcwang@wpi-aimr.tohoku.ac.jp (Z.W.).

1. Validation of potentials and nanoindentation model

1.1 Validation of potentials

To validate the potentials adopted, we calculated some fundamental physical properties of Cu-Ni alloys and compared them with the results obtained with first principles calculations and/or experiment data (Table S1 and Table S2). It can be seen in Table S1 and S2 that the parameters for Cu-Ni system potentials can well reproduce the basic physic properties; therefore, they can be used to perform the nanoindentation simulations in this work. The potential between the indenter (C) and layers (Cu and Ni)was chosen as the Morse potential, which has already been used in MD simulation for indentations, and was not verified.

Table S1 Lattice constant a (Å), cohesive energy E_c(eV) and elastic constant C_{ij} (GPa) of pure Cu and pure Ni calculated with the EAM potential, in comparison with those obtained with first principles calculation and/or experiment data

	Property	EAM(present work)	First principle	Experiment
Cu	a	3.615	3.615[a]	3.615[f]
	E_c	3.54	3.54[a]	3.54[f]
	C_{11}	176.5	171[c]	168.3[b] ,176.2[d],166.1[e]
	C_{12}	128.6	122[c]	122.1[b],124.9[d],119.9[e]
	C_{44}	76.6	75.3[c]	75.7[b],81.8[d],75.6[e]
Ni	a	3.52	3.52[a]	3.52[f]
	E_c	4.45	4.45[a]	4.45[f]
	C_{11}	239.4	247[g]	248.1[b],250.8[f]
	C_{12}	141.8	148[g]	154.9[b],150[f]
	C_{44}	120.2	125[g]	124.2[b],123.5[f]

[a] *From*[1], [b] *From* [2], [c] *From*[3], [d] *From*[4], [e] *From*[5], [f] *From*[6], [g] *From*[7]

Table S2 Lattice constant a (Å), cohesive energy E_c (eV) and elastic constant C_{ij} (GPa) of Cu/Ni alloys calculated with EAM potential, in comparison with experimental data and the results obtained with first-principles calculation.

structure	property	EAM(present work)	First principle or experiment
CuNi $L1_1$	a	3.566	3.566[a]
	E_c	3.97	3.99[b]
	C_{11}	220	205[c]
	C_{12}	140	140[c]
	C_{44}	106	99[c]
Cu_3Ni L12	a	3.544	3.587[a]
	E_c	4.203	3.694[b]
	C_{11}	188	186.9[c]
	C_{12}	104	131.1[c]
	C_{44}	98	87.8[c]
CuNi L10	a	3.566	3.566[a]
	E_c	3.968	3.95[b]

[a] *From*[8], [b] *From*[7], [c] *From*[9]

1.2. Validation of nanoindentation model

We also performed nanoindentation simulation on Cu (111) and Ni (111) surface, and the force-depth (P-h) curves were presented in Figure S1 (a), where we can see that the force is positive at h = 0 Å, which should be ascribed to the repulsion between the indenter and specimen atoms when their distance is smaller than the equilibrium one. The same phenomena have also been found in the simulations by other researchers [10,11], where a real pairwise potential (LJ or Morse potential) was used to describe the force between the indenter and the films, rather than using a repulsive potential ($V(r_{ij}) = K(R-r_{ij})^2$). According to the Hertz contact theory, P can be expressed as $P = \frac{4}{3} E R^{1/2} H^{3/2}$, where E, R and H are the reduced modulus, indenter radius and indentation depth. Based on this theory, P equals zero at H=0. Therefore, the result by MD simulation does not match well with the Hertz result well at initial indentaion.

To compare our result with that by the Hertz theory, a new depth, $h'=h+r_c$, was introduced to make P=0 at H=0 (Hertz theory), and the P-h' curves was given in Figure S1(b).

From Fig. S1(b), we can find that at the initial stage (0 Å<h'<1.8 Å), the curves obtained by MD simulations cannot match the Hertz result well, which should be ascribed to the following reasons: (1) the effect of pair potential between the indenter and the film; (2) the indenter is not an perfectly smooth sphere, but consists of many C atoms. However, at the following stage, the MD result can match the Hertz result better. And the fitted Young's muduli of Cu and Ni are 225 GPa and 330 GPa, respectively, larger than the other's results [12], which could be ascribed to the following factors: (1) The effects of potential between film atoms. The fitted parameters E is different with different potentials, which, however, less affects the deformation mechanism [12]; (2) The effect of high indentation velocity. More discussions are shown in Figure S1.

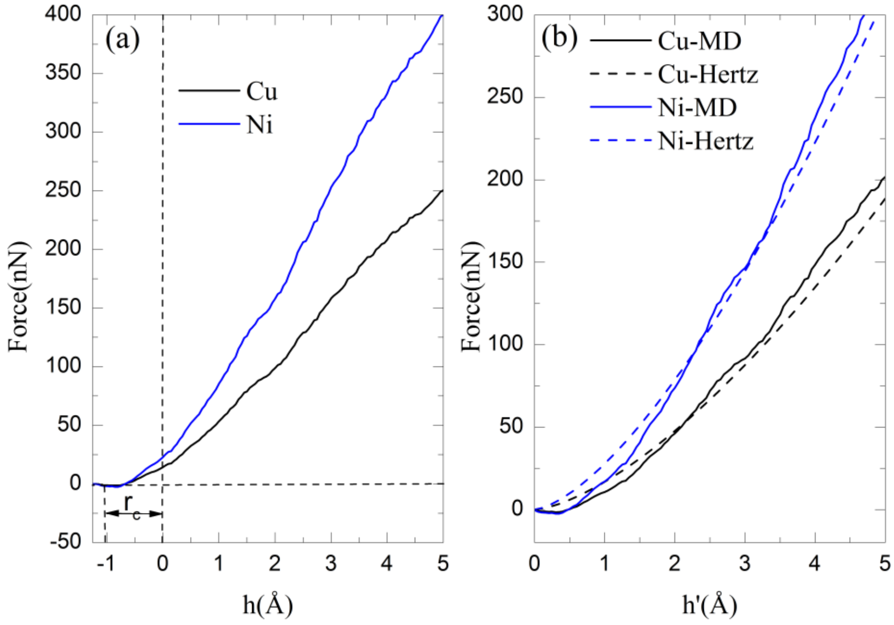

Figure S1. (a) *P-h* curves for pure Cu and Ni film; (b) *P-h'* curves for pure Cu and Ni film compared with Hertz theory.

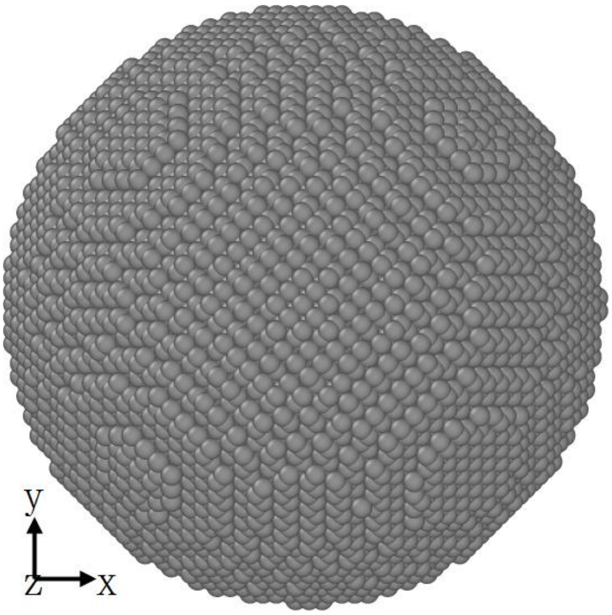

Figure S2. Surface morphography of indenter.

To further investigate the difference between result using the indenter consisting of C atoms and that with an perfectly smooth sphere, we performed more nanoindentation simulations on Cu and Ni films with a repulsive potential ($V(r_{ij}) = K(R-r_{ij})^2$ at different indentation speeds of 50m/s, 10m/s and 1m/s. Figure S3 shows the *P-h* curves of pure Cu and pure Ni films at different indentation speeds, *v*, where one can see that *P*=0 at *h*=0. We also fitted these curves with Hertz theory, and the fitted parameters *E* for Cu are 353 GPa, 249 GPa and 215 GPa corresponding to *v* =50 m/s, 10m/s and 1m/s, respectively, indicating that higher indentation speed results in higher indentation force and higher "reduced mudulus" *E*. And the *E* obtained at *v* =1 m/s can match the theoretical solution better than the simulation results of indentation at higher speeds [12]. The similar results and the tendency about the fitted parameters *E* for Ni can be found in Figure S3 (b). The depths at the first peak in the *P-h* curves of Cu at *v* =50 m/s, 10m/s and 1m/s appear at *h* =3.47 Å, 3.72 Å and 3.98 Å, respectively, which are very close to each other. It indicates that the effects of the speed range (*v* =1 m/s to *v* =50 m/s) are insufficient for the study of the deformation mechanisms of the films under nanoindentation. However, the MD simulation of the indentation lower indentation speed will take much more computational time. For example, the computational time at *v* = 1m/s would take 50 times that at *v* =50 m/s. Considering the computational efficiency and the limitation of computation capability, the indentation speeds ranging from 10m/s to 100m/s are commonly chosen to perform MD simulations [13-16]. That was why *v* = 50 m/s was chosen for the MD simulations in this work.

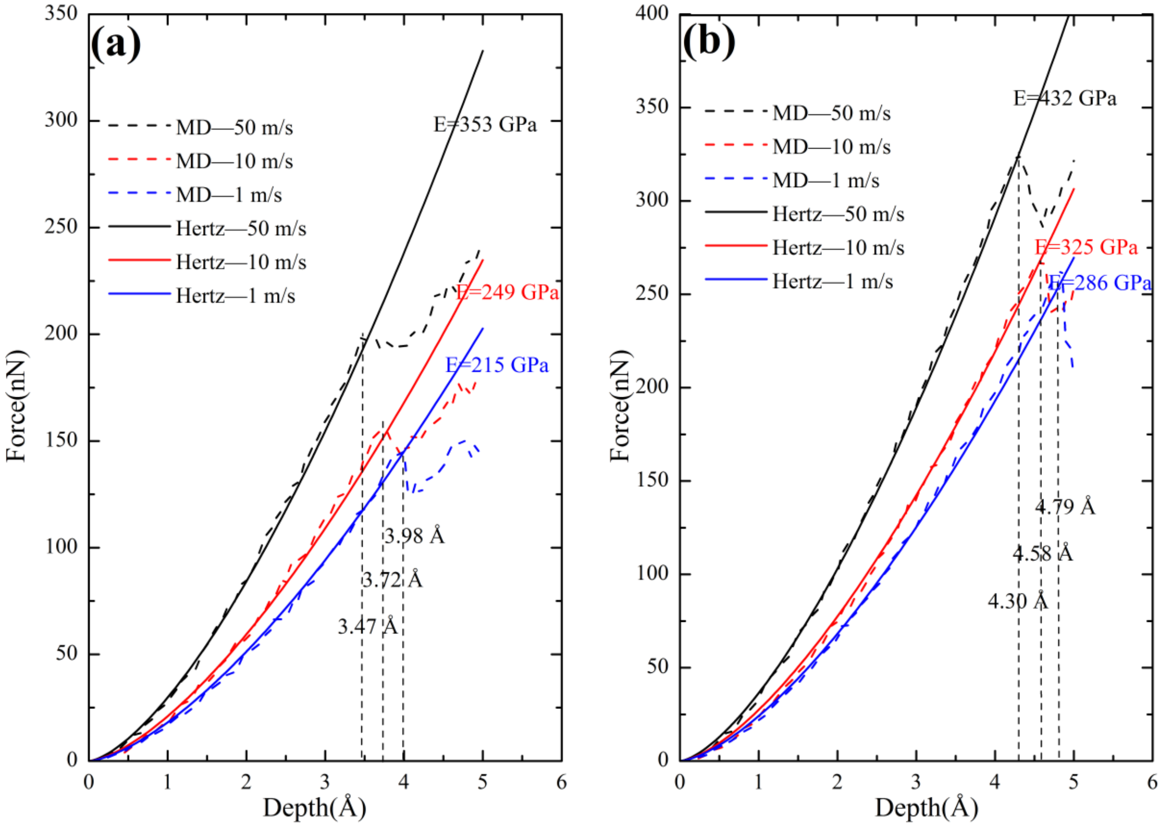

Figure S3. *P*-*h* curves of (a) pure Cu film, and (b) pure Ni film at v =50 m/s, 10m/s and 1m/s, solutions with Hertz theory are shown for comparison

2. Local *P-h* curves near the Point α and Point β

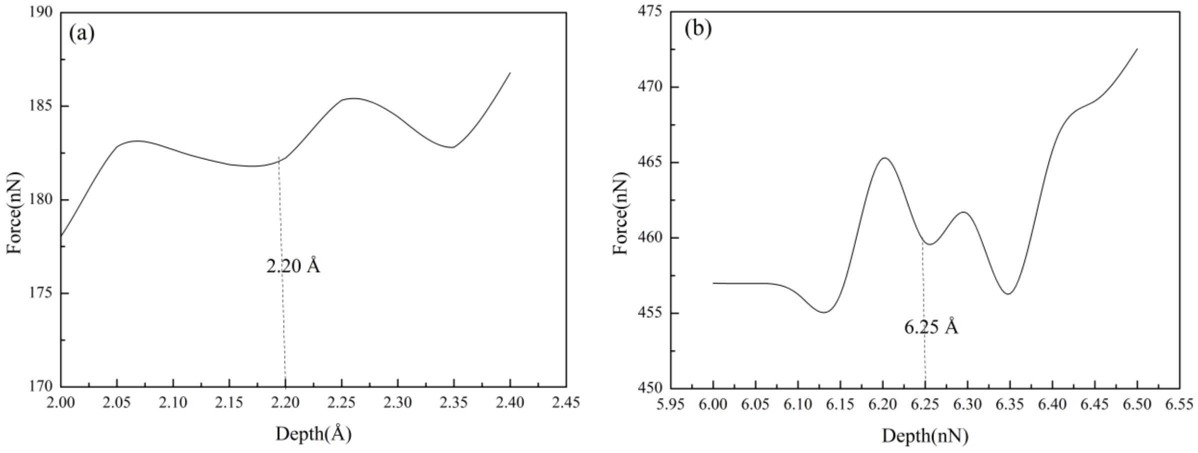

Figure S4. Local *P*-*h* curves in the vicinity of (a) Point α, and (b) Point β.

3. Initial configurations of two additional samples

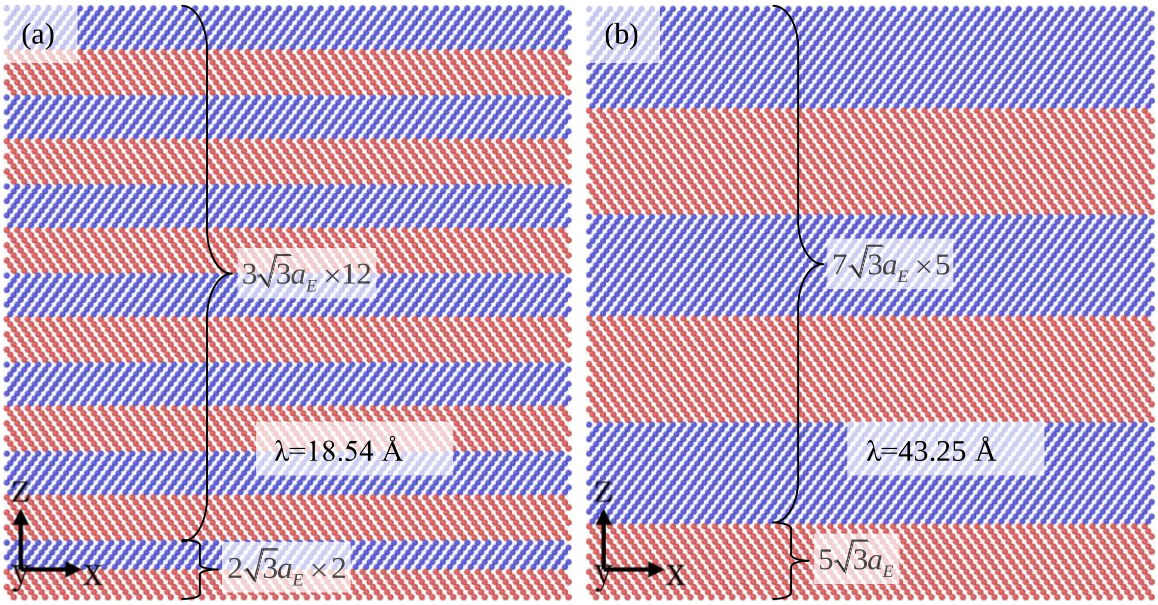

Figure S5. Initial configurations of samples viewed on X-Z plane with different twin thichness: (a) $3\sqrt{3}a_E$ (18.54 Å) and (b) $7\sqrt{3}a_E$ (43.25 Å). Red and blue balls represent Cu and Ni atoms, respectively.

4. Nanoindentation simulations with larger indenter of 50 Å in radius

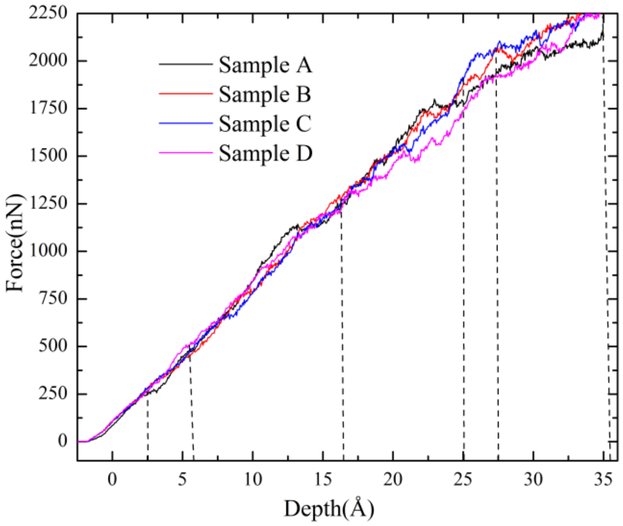

Figure S6. *P-h* curves of samples with various twin thicknesses indented with spherical indenter of 50 Å in radius.

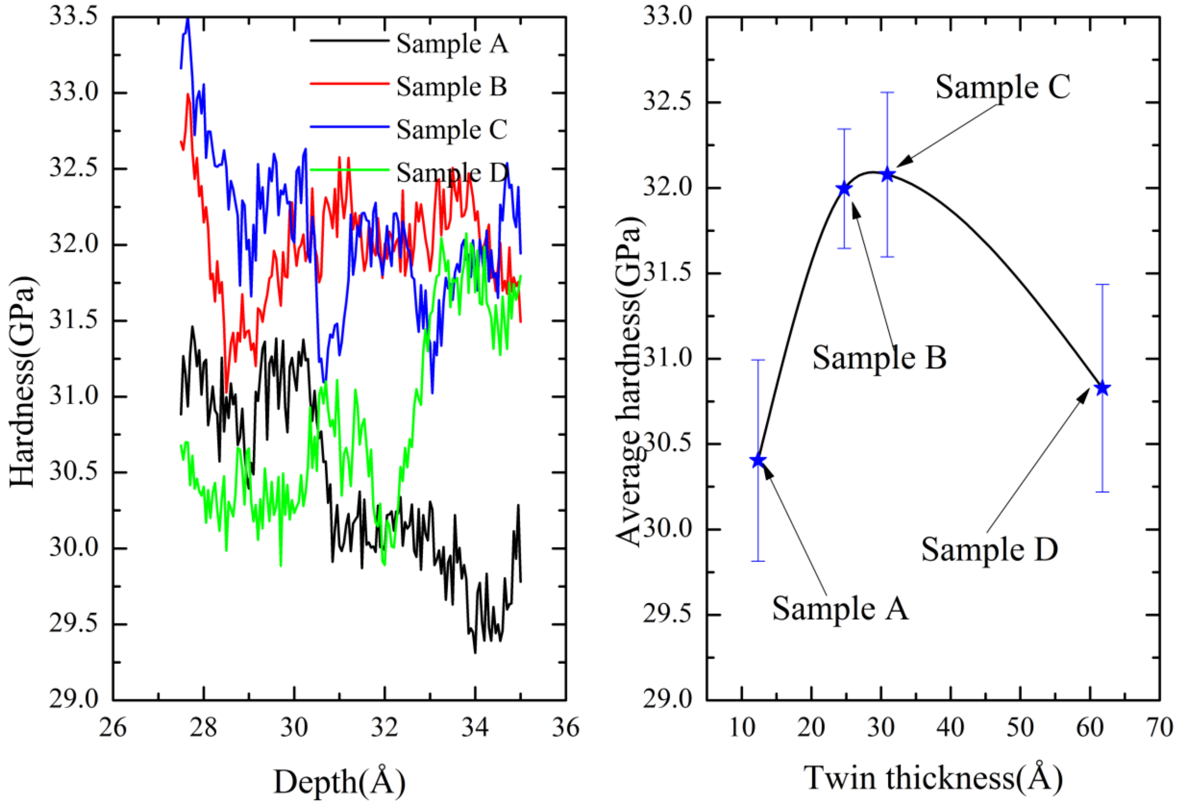

Figure S7. Variation of hardness against *h* and *λ* for the samples with indenter of 50 A in radius: (a) *H*-*h* curve, and (b) *H*-*λ* curve.

5. Nanoindentation simulations at lower indentation speed of 10 m/s

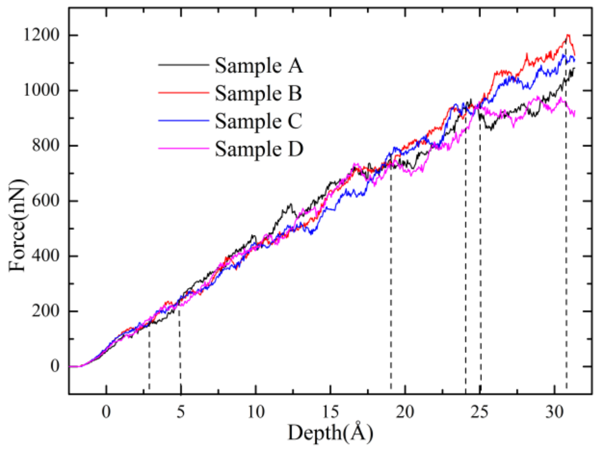

Figure S8. *P*-*h* curves of the samples with various twin thicknesses under the indentation velocity of 10 m/s with indenter size of 40 Å in radius.

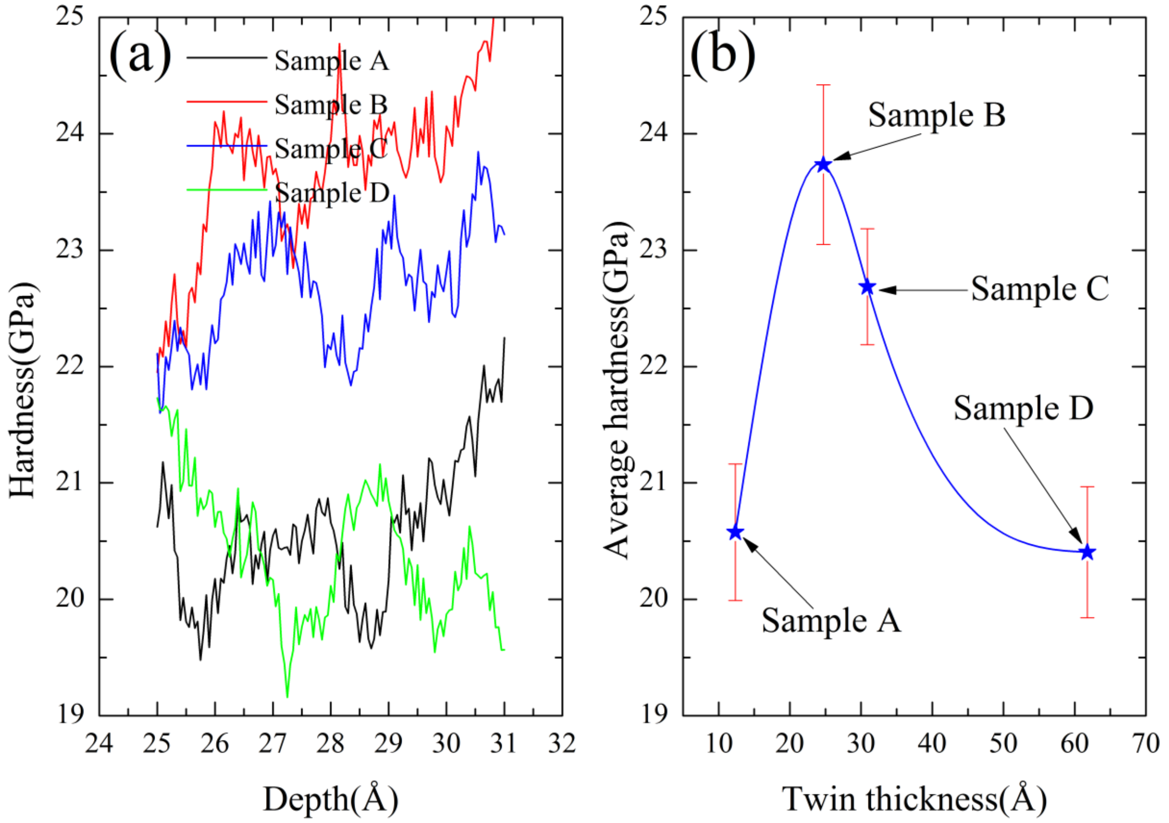

Figure S9. Variation of harness against *h* and λ for the samples under the indentation velocity of 10 m/s with indenter size of 40 Å in radius: (a) *H-h* and (b) H-λ curve.

References

1 Zhang, J.-M., Xin, H., Zhang, Y. & Xu, K.-W. Atomic-Scale Calculation of the Energy for the Cu/Ni Interface. *The Journal of Physical Chemistry C* **113**, 12272-12276, (2009).

2 Shuman, D. C. & Sen, B. Complex formation thermodynamics of some metal(II) phenyl-2-pyridylketoximates. *Analytica Chimica Acta* **33**, 487-496, (1965).

3 Bercegeay, C. & Bernard, S. First-principles equations of state and elastic properties of seven metals. *Physical Review B* **72**, 214101, (2005).

4 Overton, W. C. & Gaffney, J. Temperature Variation of the Elastic Constants of Cubic Elements. I. Copper. *Physical Review* **98**, 969-977, (1955).

5 Hiki, Y. & Granato, A. V. Anharmonicity in Noble Metals - Higher Order Elastic

Constants. *Physical Review* **144**, 411-&, (1966).

6 G. Simmons, H. W. *Single Crystal Elastic Constant and Calculated Aggregate Properties*. (MIT Press, 1971).

7 Onat, B. & Durukanoglu, S. An optimized interatomic potential for Cu-Ni alloys with the embedded-atom method. *Journal of physics. Condensed matter : an Institute of Physics journal* **26**, 035404, (2014).

8 Clarke, J. K. A. & Spooner, T. A. The formation of homogeneous copper - nickel alloy films in ultra-high vacuum. *Journal of Physics D: Applied Physics* **4**, 1196 (1971).

9 Epstein, S. G. & Carlson, O. N. The elastic constants of nickel-copper alloy single crystals. *Acta Metallurgica* **13**, 487-491, (1965).

10 Fu, T. *et al.* Molecular dynamics simulation of deformation twin in rocksalt vanadium nitride. *Journal of Alloys and Compounds* **675**, 128-133, (2016).

11 Zhu, P. Z. & Fang, F. Z. Molecular dynamics simulations of nanoindentation of monocrystalline germanium. *Applied Physics A* **108**, 415-421, (2012).

12 Zhu, T. Predictive modeling of nanoindentation-induced homogeneous dislocation nucleation in copper. *Journal of the Mechanics and Physics of Solids* **52**, 691-724, (2004).

13 Fang, T.-H., Chang, W.-Y. & Huang, J.-J. Dynamic characteristics of nanoindentation using atomistic simulation. *Acta Materialia* **57**, 3341-3348, (2009).

14 Hasnaoui, A., Derlet, P. M. & Van Swygenhoven, H. Interaction between dislocations and grain boundaries under an indenter – a molecular dynamics simulation. *Acta Materialia* **52**, 2251-2258, (2004).

15 Yang, B. *et al.* Atomistic simulation of nanoindentation on incipient plasticity and dislocation evolution in γ/γ′ phase with interface and void. *Computational Materials Science* **114**, 172-177, (2016).

16 Alhafez, I. A., Ruestes, C. J., Gao, Y. & Urbassek, H. M. Nanoindentation of hcp metals: a comparative simulation study of the evolution of dislocation networks. *Nanotechnology* **27**, 045706, (2016).
